# Test, Treat, Repopulate™ method reduces red complex bacteria and stabilizes the oral microbiome

**DOI:** 10.3389/fdmed.2026.1839034

**Published:** 2026-05-29

**Authors:** Rachelle Beattie McAliley, Lora Hooper, Kami Hoss, David J. Vigerust

**Affiliations:** 1 ProBiora Health, LLC, Sarasota, FL, United States; 2 SalivaHacker™, Parrish, FL, United States; 3 SuperMouth, LLC, San Diego, CA, United States; 4 SimplyTest, LLC, Huntsville, AL, United States; 5 Woody L. Hunt School of Dental Medicine, Texas Tech University Health Sciences, El Paso, TX, United States

**Keywords:** dental caries, oral microbiome, periodontitis, probiotics, salivary diagnostics

## Abstract

Despite advances in dental hygiene and clinical interventions, preventable conditions such as tooth decay and gum disease remain widespread public health concerns. Recent research implicates complex, polymicrobial interactions as central drivers of disease pathogenesis, which often limits the effectiveness of traditional therapeutic approaches. Here, researchers evaluate the effectiveness of the Test, Treat, Repopulate method, a multi-phase oral health protocol combining scaling and root planing, targeted antibiotic therapy, use of a pH-balanced, prebiotic containing toothpaste and rinse, and daily oral probiotic use on the health of the oral microbiome. Retrospective analysis of RT-qPCR salivary diagnostic data from 38 de-identified U.S. patients, collected before and after treatment during routine care, demonstrated significant and sustained reductions in key oral pathogens following the combined treatment approach. Seven bacterial species were significantly reduced post-treatment, including *Treponema denticola*, *Tannerella forsythia*, *Prevotella intermedia*, *Campylobacter rectus*, *Fusobacterium nucleatum*, *Fusobacterium nucleatum* subsp*. animalis*, and *Streptococcus mutans*. Additional pathogens, including *Aggregatibacter actinomycetemcomitans* and *Porphyromonas gingivalis*, were also reduced. The protocol had minimal impact on the fungal species *Candida albicans*, highlighting an area for potential optimization. Additionally, four viral targets were assessed pre- and post-protocol, making this one of the first studies to evaluate a dental intervention's effects on oral viruses. Comparison of the cohort with 35,570 pre-treatment salivary diagnostic tests across the United States shows that baseline pathogen distributions reflect national patterns, supporting the generalizability of the protocol. A structured protocol for patients with elevated red complex bacteria is detailed, providing clinicians with a data-driven, reproducible framework for precision oral healthcare. These findings underscore the potential for microbiome-focused interventions to achieve measurable, lasting improvements in oral health across diverse populations.

## 1 Introduction

Periodontal disease and tooth decay remain significant global health concerns, affecting both oral and systemic health (1, 2). These conditions arise from dysbiosis in the oral microbiome, where pathogenic species outcompete commensal bacteria, leading to inflammation, tissue destruction, and ultimately tooth loss (3, 4). Despite widespread use of mechanical debridement and antimicrobial therapies, long-term disease control remains challenging, underscoring the need for strategies that address microbial ecology rather than transient pathogen suppression.

Among the organisms most strongly associated with periodontal disease are the red complex bacteria, *Porphyromonas gingivalis*, *Treponema denticola*, and *Tannerella forsythia*, which act synergistically to disrupt host immune responses and promote inflammatory tissue damage (5–7). In addition to the red complex bacteria, other bacterial taxa, including *Prevotella intermedia*, *Campylobacter rectus*, and *Fusobacterium nucleatum* also contribute to disease progression and biofilm maturation (8) (Table 1). While traditional therapies including scaling and root planing and systemic antibiotics can transiently reduce bacterial burden, recolonization by pathogenic species frequently occurs, limiting long-term efficacy (9).

**Table 1:** Salivary diagnostic targets measured, the oral health target category to which they belong, and the microbial classification type of each target.

Species	Oral health target category	Target type
*Aggregatibacter actinomycetemcomitans*	Purple complex	Bacterium
*Prophyromonas gingivalis*	Red complex	Bacterium
*Tannerella forsythia*	Red complex	Bacterium
*Treponema denticola*	Red complex	Bacterium
*Prevotella intermedia*	Orange complex	Bacterium
*Campylobacter rectus*	Orange complex	Bacterium
*Fusobacterium nucleatum*	Orange complex	Bacterium
*Fusobacterium nucleatum* subsp. *animalis*	Orange complex	Bacterium
*Eikenella corrodens*	Green complex	Bacterium
*Streptococcus mutans*	Caries Risk	Bacterium
*Candida albicans*	Fungal	Fungus
Herpes simplex virus 1 (HSV-1)	Viral	Virus
Herpes simplex virus 2 (HSV-2)	Viral	Virus
Cytomegalovirus	Viral	Virus
Epstein Barr Virus	Viral	Virus
*Streptococcus sanguinis*	Health Indicator	Bacterium

In addition to bacteria, fungi and viruses also play important roles in the oral cavity. *Candida albicans* is frequently detected in the oral cavity and may interact with bacterial species to influence biofilm structure and pathogenicity (10, 11). Oral viruses, including herpesviruses, have been detected in periodontal lesions and may exacerbate inflammatory responses, yet they are rarely evaluated in clinical dental protocols (12–15). As a result, most periodontal interventions remain bacteria-centric, leaving other microbial kingdoms underexplored in both diagnostics and treatment assessment.

Recent advances in salivary diagnostics have enabled high-resolution, quantitative assessment of oral pathogens across diverse patient populations using noninvasive sampling and molecular techniques such as quantitative PCR (16, 17). Salivary diagnostic testing enables clinicians to identify and quantify both harmful and commensal oral bacteria, fungi, and viruses through analysis of patient saliva using a simple swish and spit test. These tools enable clinicians to characterize baseline microbial profiles, monitor treatment response, and objectively assess changes over time. Importantly, large-scale salivary diagnostic datasets provide an opportunity to contextualize individual patient results within population-level microbial distributions, supporting broader inference and protocol generalizability.

Here, the researchers evaluate the Test, Treat, Repopulate protocol, a diagnostic-guided, multi-phase approach combining scaling and root planing, targeted antibiotic therapy, a stuctured pH-balanced oral care regimen using pH-balanced, prebiotic-containing toothpaste and rinse, and daily oral probiotic use. Outcomes from a cohort of 38 individuals are assessed using salivary diagnostics before and after treatment and compared to 35,570 pre-treatment salivary diagnostic tests collected across the United States. This comparison allows evaluation of whether the study cohort reflects national pathogen distributions and supports wider applicability of the protocol. In addition to bacterial and fungal outcomes, this study assesses four viral targets, representing one of the first evaluations of a dental intervention's effects on viral presence in the oral cavity.

## 2 Materials and methods

### 2.1 Study design and data sources

All analyses were conducted using de-identified clinical salivary diagnostic data to ensure patient privacy and compliance with ethical standards. No personally identifying information was collected or analyzed. Salivary diagnostic testing was performed as a part of routine clinical assessment, and all participants provided informed consent for the use of their data in aggregated research analyses.

Pre-and post-protocol salivary diagnostic data from a total of 38 individuals were included in this retrospective study (see Supplementary Table S1 for participant details). No directly identifiable private individual information was collected, accessed, or analyzed by the investigators, and all participants provided consent for use of their data in aggregated research analyses. Because this work does not involve human subjects as defined under 45 CFR 46, formal Institutional Review Board approval is not required. The cohort was nearly evenly split between male (20, 53%) and female (18, 47%) participants with an average age of 45.4 years and were located throughout the United States. Baseline pathogen distributions from this cohort were compared to a national reference dataset comprising 35,570 pre-treatment salivary diagnostic tests collected across the United States between the dates of September 22, 2023, to October 13, 2025, which served as a population-level comparator for evaluating cohort representativeness and generalizability.

### 2.2 Salivary diagnostic testing

Human saliva specimens submitted for periodontal pathogen identification including bacterial, viral, and fungal targets, were handled as clinical human-source material under standard biosafety practices. All specimen manipulations were performed in a certified Class II biosafety cabinet, with single-container opening to reduce cross-contamination and specimen mix-up risk.

Total nucleic acids were extracted in 96-well deep-well plates using the MagMAX™ DNA Multi-Sample Ultra 2.0 Kit on a KingFisher™ Flex instrument. Enhancer solution, specimen, and Proteinase K were combined per well; external positive and negative controls were processed in parallel. Automated extraction used sequential washes (Wash Solution 1; 80% ethanol) and elution in 50 µL, with Xeno internal control added to the elution solution.

Extracted nucleic acids underwent multiplex pre-amplification (14 cycles) using TaqPath™ 1-Step RT-qPCR Master Mix (CG) and a custom TaqMan™ PreAmp primer pool. Standard curve materials and external controls were included on each plate. Pre-amplification products were diluted 1:10 in nuclease-free water before OpenArray loading. Workflow contamination controls included physical segregation of reagent setup, pre-PCR, and post-PCR areas, plus glove changes at critical seal-removal steps.

Real-time PCR was performed on custom 56-assay OpenArray® plates (PD-Metrix) loaded with the QuantStudio™ 12K Flex OpenArray® AccuFill™ system. Each OpenArray plate contains 3,072 through-holes supporting ∼33 nL reactions. AccuFill sample plates were prepared with TaqMan™ OpenArray® Real-Time PCR Master Mix and diluted pre-amplified product, then sealed and centrifuged before loading. After loading, plates were sealed within 90 s, filled with immersion fluid, plugged, and run on a QuantStudio™ 12K Flex using assay-specific TPF settings. Post-run QC imaging confirmed proper loading and plate integrity before analysis.

Run data were processed in QuantStudio™ 12K Flex software and exported to Thermo Fisher Scientific Analysis Macro v1.48 for replicate compilation by target and sample. Valid amplification criteria were: Crt ≤28, amplification score ≥1.2, Cq confidence ≥0.8, and Crt SD ≤0.5. A target was called detected when ≥2 replicate through-holes met all filters. For quantification, sample Crt values were converted to concentration using run-specific standard curves; curve fitting and concentration calculations were performed with laboratory-developed Python scripts.

### 2.3 Treatment protocol: Test, Treat, Repopulate

Baseline salivary diagnostics were used to quantify bacterial and fungal species and assess viral presence prior to treatment (Test). The specific microorganisms included in the salivary diagnostic panel were selected because their detection in saliva is significantly correlated with their detection in gingivitis, periodontitis, caries formation, oral dysbiosis, and other clinically significant oral health concerns (18–20). Results informed clinical decision-making and identified individuals with elevated red complex and associated pathogens, which are known to be directly responsible for the development and progression of severe periodontitis (19). Following baseline testing, all participants underwent scaling and root planing to mechanically disrupt supragingival and subgingival biofilms. This was followed by a 14-day course of targeted systemic antibiotics (amoxicillin and metronidazole), a regimen commonly used to reduce anaerobic periodontal pathogens (9). Upon completion of mechanical and antibiotic therapy (Treat), participants initiated a daily oral care regimen consisting of pH-balanced, prebiotic-containing toothpaste and rinse (SuperMouth®), followed by oral probiotic administration (ProBiora3®). This phase (Repopulate) aimed to reintroduce health-associated *Streptococcus* species and support competitive inhibition of pathogenic bacteria, consistent with ecological models of biofilm stabilization (4, 21, 22).

Follow-up salivary diagnostics were collected at multiple intervals ranging from 25 days to 274 days post-treatment; this timing was not standardized across participants to allow for their routinely scheduled exams. Primary outcomes included changes in bacterial abundance relative to baseline, with secondary outcomes assessing fungal presence and viral detection status. Results were evaluated within individuals over time and contextualized against national baseline distributions to assess the broader applicability of the protocol.

### 2.4 Data analysis and interpretation

All oral microorganisms measured in this study were assessed using RT-QPCR as described in Methods Section 2.2 above. Bacterial outcomes were evaluated based on changes in relative abundance measured by qPCR, with statistical significance determined using paired pre- and post-treatment comparisons (GraphPad Prism version 10.6.1). For statistical analyses, targets that were coded as nondetects (below the limit of detection) were imputed using the nondetects package in R version 4.5.2 (23). Viral outcomes were assessed descriptively based on presence or absence. Comparisons between the study cohort and the national dataset focused on baseline pathogen prevalence patterns to assess similarity and generalizability of findings across U.S. patient populations.

## 3 Results

**Figure 1:**
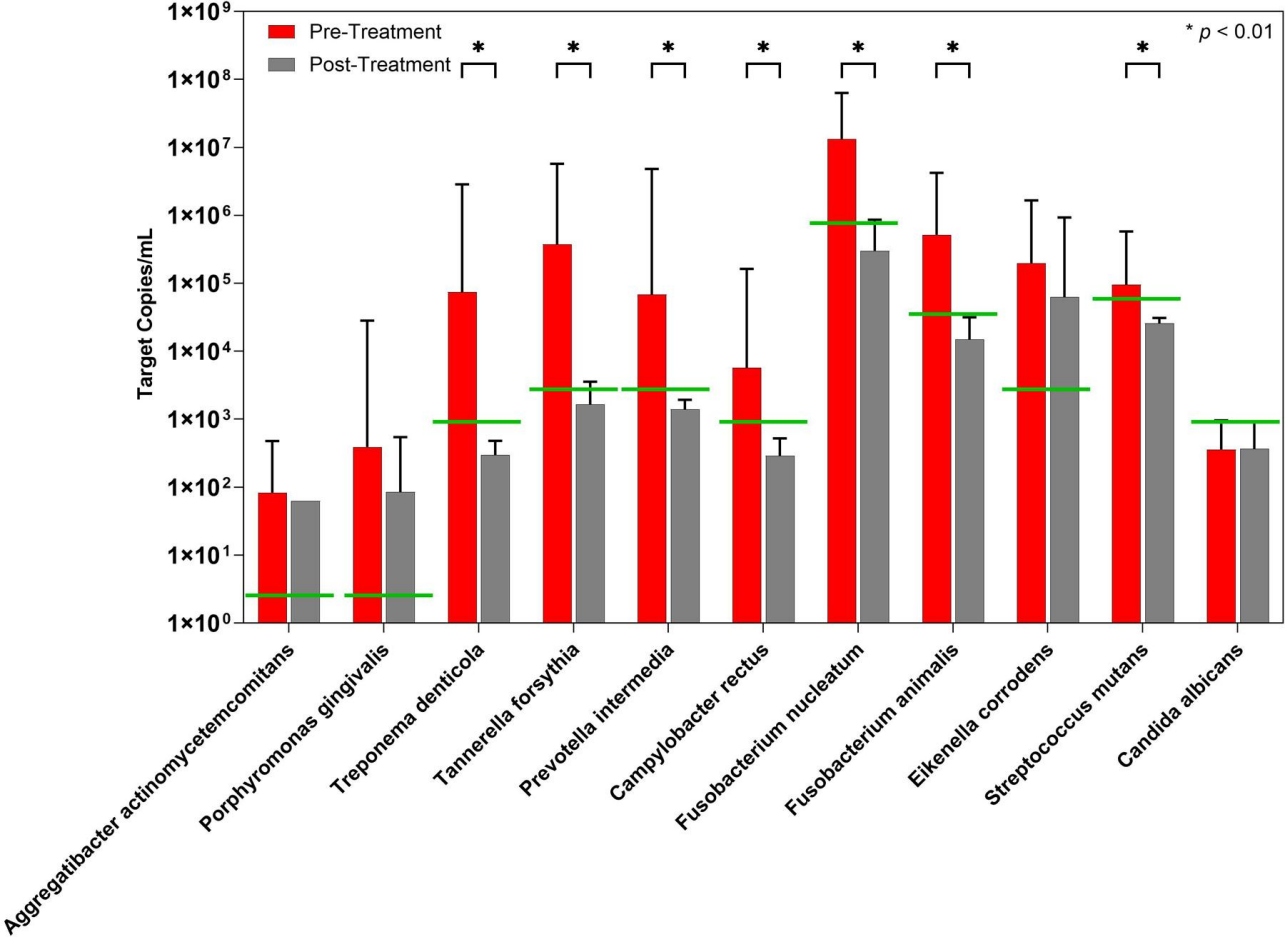
Target copies/mL measured at baseline and at the first follow-up visit following implementation of the Test, Treat, Repopulate protocol. Follow-up saliva testing was conducted between 25 and 275 days post-treatment, with a mean retest interval of 70 days. Targets exhibiting statistically significant reductions after protocol implementation are denoted by an asterisk (*; *p* < 0.01). Green reference lines indicate the threshold below which each target is classified as low abundance in the oral cavity, representing the desired benchmark for oral health.

Salivary diagnostic analysis revealed robust reductions in multiple clinically relevant oral pathogens following completion of the multi-phase protocol. Among the 38 individuals analyzed, seven bacterial species exhibited statistically significant reductions post-treatment (Figure 1). These included the periodontal pathogens *Treponema denticola*, *Tannerella forsythia*, *Prevotella intermedia*, *Campylobacter rectus*, *Fusobacterium nucleatum*, and *Fusobacterium nucleatum* subsp. *animalis*, as well as the caries-associated species *Streptococcus mutans*. Several additional pathogens demonstrated consistent downward trends that did not reach statistical significance, including *Aggregatibacter actinomycetemcomitans* and *Porphyromonas gingivalis* (Figure 1). While reductions in these species were observed in a subset of individuals, inter-individual variability limited statistical power.

**Figure 2:**
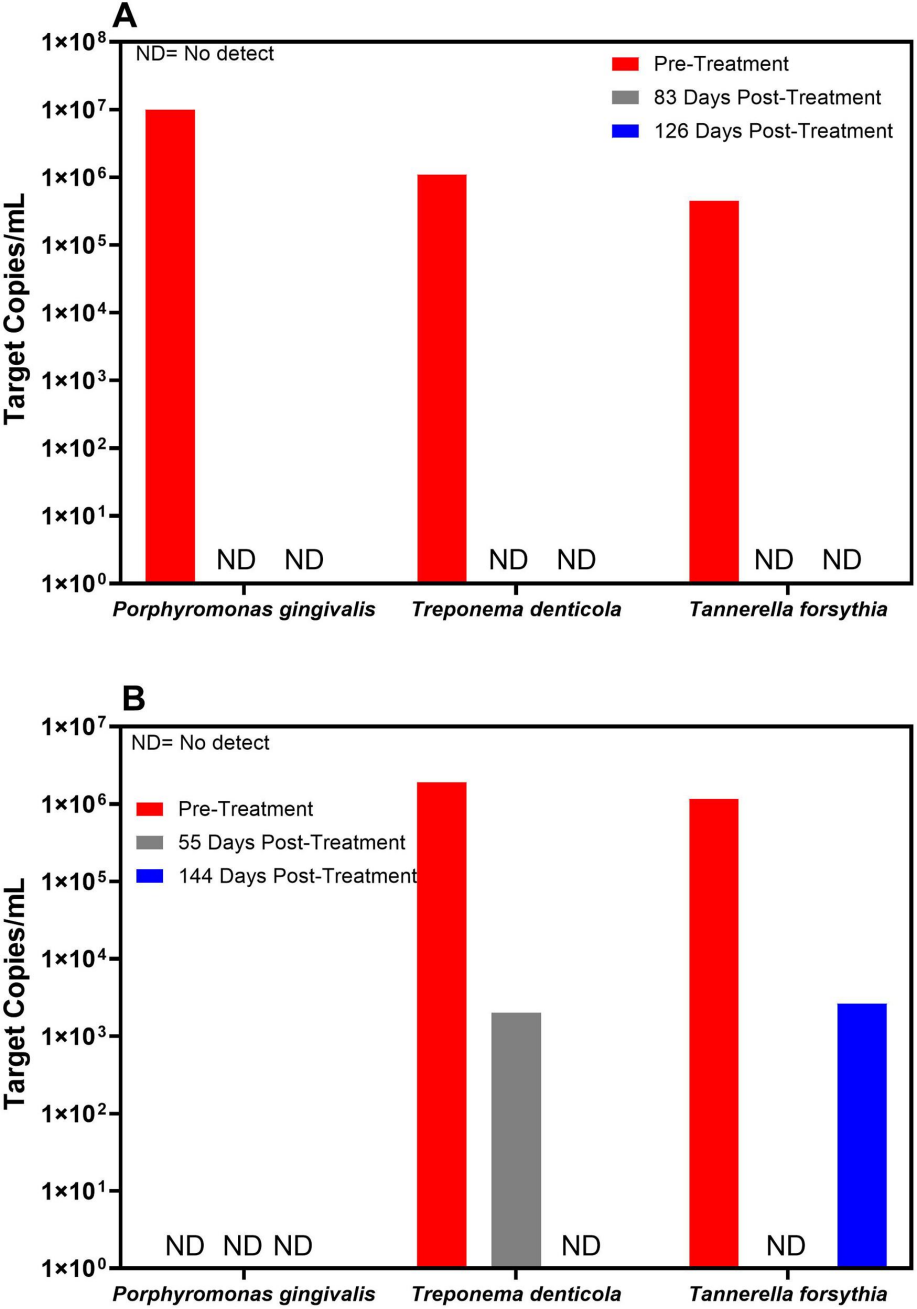
Target copies/mL for two patients **(A, B)** that participated in continued monitoring after the implementation of the Test, Treat, Repopulate protocol. Non-detects are displayed as ND.

Across the cohort, total pathogenic bacterial load decreased by more than 90% relative to baseline measurements. Two patient case-studies demonstrated that red complex bacteria can continue to be suppressed without additional mechanical or antibiotic intervention when individuals adhere to daily probiotic and rinse use, supporting the durability of microbiome modulation achieved through this approach (Figure 2).

In contrast to bacterial outcomes, the protocol did not result in significant reduction of the fungal species *Candida albicans*, indicating that this intervention is primarily effective against bacterial members of the oral microbiome and may require modification for fungal dysbiosis (Figure 1). Additionally, the presence and absence of the four viral targets was not significantly changed after completion of the protocol (Table 2).

**Table 2:** Presence and absence data of the four viral targets for 38 study participants before and after the Test, Treat, Repopulate protocol intervention.

Study participant	Herpes simplex virus 1 (HSV-1)		Herpes simplex virus 2 (HSV-2)		Cytomegalovirus		Epstein Barr virus	
	Before	After	Before	After	Before	After	Before	After
1	-	-	-	-	-	-	-	-
2	-	-	-	-	-	-	+	+
3	-	-	-	-	-	-	-	-
4	-	-	-	-	-	-	-	+
5	-	-	-	-	-	-	+	+
6	-	-	-	-	-	-	-	+
7	-	-	-	-	-	-	+	+
8	-	-	-	-	-	-	-	-
9	-	-	-	-	-	-	-	-
10	-	-	-	-	-	-	-	-
11	-	-	-	-	-	-	-	-
12	-	-	-	-	-	-	+	-
13	-	-	-	-	-	-	+	-
14	-	-	-	-	+	-	-	+
15	-	-	-	-	-	-	+	-
16	-	-	-	-	+	-	-	+
17	-	-	-	-	-	-	-	+
18	-	-	-	-	-	-	-	-
19	-	-	-	-	-	-	+	+
20	-	-	-	-	-	-	-	-
21	-	-	-	-	+	+	-	-
22	-	-	-	-	-	-	-	-
23	-	-	-	-	-	-	-	-
24	-	-	-	-	-	-	+	-
25	-	-	-	-	-	-	-	+
26	-	-	-	-	-	-	-	-
27	-	-	-	-	-	+	-	-
28	-	-	-	-	-	+	-	-
29	-	-	-	-	+	-	-	-
30	+	-	-	-	-	-	-	-
31	-	-	-	-	+	+	-	-
32	-	-	-	-	+	+	-	-
33	-	+	-	-	-	-	-	-
34	-	-	-	-	+	+	-	-
35	-	-	-	-	+	-	-	-
36	-	-	-	-	-	+	-	-
37	-	-	-	-	+	+	-	-
38	-	-	-	-	-	-	-	-

A “+” symbol and grey shading indicates the target was detected while a “-” symbol indicates the target was absent from the sample.

Baseline salivary diagnostics demonstrated that the 38-individual study cohort exhibited pathogen detection patterns largely consistent with those observed in a national reference dataset of 35,570 pre-treatment samples (Table 3). Detection frequencies for red complex bacteria were comparable between cohorts, with *Treponema denticola* detected in 76.3% of the study cohort vs. 61.8% nationally and *Tannerella forsythia* detected in 89.5% vs. 79.1%, respectively. *Porphyromonas gingivalis* showed lower overall prevalence in the study cohort but followed a similar distribution pattern (15.8% vs. 20.9%). Orange complex species, including *Prevotella intermedia*, *Campylobacter rectus*, and *Fusobacterium nucleatum*, were prevalent in both datasets, with *F. nucleatum* detected in nearly all individuals (100.0% in the study cohort; 97.2% nationally).

**Table 3:** Prevalence of microbial targets in the oral microbiome in the study cohort of 38 individuals and the overall population at baseline (prior to the protocol intervention).

Species	% of cohort with detectable target	% of overall population with detectable target
*Aggregatibacter actinomycetemcomitan*s	2.6%	4.0%
*Porphyromonas gingivalis*	15.8%	20.9%
*Treponema denticola*	76.3%	61.8%
*Tannerella forsythia*	89.5%	79.1%
*Prevotella intermedia*	50.0%	42.8%
*Campylobacter rectus*	55.3%	37.4%
*Fusobacterium nucleatum*	100.0%	97.2%
*Fusobacterium nucleatum* subsp. *animalis*	92.1%	86.1%
*Eikenella corrodens*	97.4%	90.7%
*Streptococcus mutans*	39.5%	39.4%
*Candida albicans*	13.2%	9.3%
Herpes simplex virus 1 (HSV-1)	2.6%	4.6%
Herpes simplex virus 2 (HSV-2)	0.0%	0.1%
Cytomegalovirus	23.7%	0.8%
Epstein Barr virus	21.1%	42.5%
*Streptococcus sanguinis*	100.0%	97.0%

Caries-associated and health-associated species showed close alignment across cohorts, with *Streptococcus mutans* detected in 39.5% of the study cohort and 39.4% nationally, and *Streptococcus sanguinis* present in 100.0% and 97.0%, respectively. Detection of *Candida albicans* was relatively low in both groups (13.2% vs. 9.3%). Viral targets were detected at low to moderate frequencies, with HSV-1 present in 2.6% of the study cohort compared to 4.6% nationally, HSV-2 rarely detected in either group, and Epstein–Barr virus observed in both datasets, though with greater variability. Collectively, these data indicate that the baseline microbial profile of the 38-individual cohort reflects broader U.S. population patterns, while differences likely reflect the modest cohort size, regional or practice-level case mix, and heterogeneity in periodontal disease severity or prior treatment history.

## 4 Discussion

This study demonstrates that the Test, Treat, Repopulate method is an effective, diagnostic-guided approach for reducing key periodontal and caries-associated bacterial pathogens while supporting stabilization of the oral microbiome. Significant reductions were observed across multiple red and orange complex species, including *Treponema denticola* and *Tannerella forsythia*, organisms strongly associated with periodontal inflammation and disease progression (5, 6). Importantly, baseline pathogen distributions in the study cohort closely mirrored those observed in a national dataset of 35,570 individuals, supporting the applicability of this protocol across diverse patient populations in the United States.

From a clinical perspective, the Test, Treat, Repopulate method aligns well with existing periodontal workflows and can be readily incorporated into routine dental practice. Salivary diagnostic testing provides an objective, noninvasive tool to evaluate patients based on microbial risk, enabling clinicians to move beyond symptom-based treatment toward precision oral healthcare (16, 24). Baseline testing identifies patients with elevated red complex and associated pathogens who may benefit most from adjunctive antimicrobial and probiotic strategies, while follow-up testing allows clinicians to document treatment response and reinforce patient compliance.

The treatment phase of the protocol leverages established periodontal interventions, including scaling and root planing and short-term systemic antibiotic therapy, which remain standard of care for patients with advanced oral microbial dysbiosis (9). The addition of a structured repopulation phase using a pH-balanced, prebiotic-containing toothpaste and rinse, along with oral probiotics addreses a critical gap in conventional care by promoting recolonization with health-associated bacteria. Ecological models of the oral biofilm suggest that sustained microbial balance requires not only pathogen reduction but also competitive exclusion and stabilization by commensal species (3, 4). The maintenance of reduced pathogen levels for up to 12 months in compliant individuals supports the clinical relevance of this approach.

While the protocol was effective against bacterial pathogens, it had minimal impact on *Candida albicans*, indicating that fungal dysbiosis may require additional or alternative interventions. This finding is consistent with prior work demonstrating that fungal–bacterial interactions contribute to biofilm resilience and may not be adequately addressed by antibacterial strategies alone (10).

Similarly, although viral targets were assessed, no significant changes in viral presence were observed. However, the appearance of EBV or CMV detection after treatment in some individuals warrants careful interpretation. Post-treatment detection of Epstein–Barr virus (EBV) in periodontal patients is biologically plausible because EBV is commonly shed in saliva in healthy carriers and is also associated with inflamed periodontal tissues and periodontal pockets in periodontitis. During active disease, EBV may be present in gingival epithelial cells, B lymphocytes, and plasma-cell–rich inflammatory infiltrates, and local periodontal conditions may favor lytic viral activation. Mechanical periodontal instrumentation may therefore transiently increase the amount of detectable EBV DNA in saliva or sampled periodontal sites by disrupting inflamed tissues that already harbor virus. Importantly, this should not be interpreted as evidence that periodontal treatment newly causes EBV infection. Rather, the overall literature suggests that periodontal therapy tends to reduce EBV burden over time by decreasing inflammation and altering the local microbial environment that supports EBV activation. Clinical studies have shown significant reductions in EBV DNA after initial periodontal treatment, and mechanistic studies indicate that bacterial metabolites such as butyrate may contribute to EBV reactivation in diseased periodontal tissues (25–30).

A similar framework applies to human cytomegalovirus (HCMV/CMV), although the biology differs in important ways. CMV has been linked to periodontitis lesions and may be detected in saliva and periodontal sites of affected patients, with evidence suggesting that periodontitis lesions can be a major source of salivary CMV. As with EBV, periodontal instrumentation of inflamed tissues may transiently increase detectable CMV DNA shortly after treatment because the procedure disturbs a preexisting viral reservoir. However, CMV latency and reactivation are more closely tied to myeloid-lineage cells, inflammatory signaling, and host immune context, and salivary CMV may also reflect replication related to salivary-gland biology rather than periodontal tissue alone. Even so, the available periodontal-treatment literature indicates that successful therapy is associated with substantial reductions in CMV copy counts in both saliva and periodontal sites. Thus, post-treatment CMV positivity is most reasonably interpreted as transient detection from previously infected inflamed tissues, whereas sustained or repeated CMV positivity may warrant greater attention to host inflammatory status or immune compromise (28, 31–33).

Comparison of the study cohort with a large national reference dataset provides useful context for interpreting baseline microbial patterns but also highlights important differences that should be considered. While the overall distribution of key oral pathogens was broadly similar, the study cohort demonstrated higher detection frequencies for several periodontal-associated organisms, including *Treponema denticola*, *Tannerella forsythia*, and *Campylobacter rectus*, as well as cytomegalovirus, whereas *Porphyromonas gingivalis* and Epstein–Barr virus were detected less frequently than in the national population. These differences likely reflect a combination of factors, including the relatively small cohort size, which increases sensitivity to individual variation; the clinical selection of patients undergoing this specific treatment protocol, and regional or practice-level differences in patient populations. Additionally, variability in periodontal disease severity, prior treatment history, and host factors may contribute to shifts in detectable microbial patterns. As such, the national comparison is best interpreted as providing a broad contextual benchmark.

Several limitations should be considered. The cohort size was modest, and the pre-post design did not include a control group; therefore, the observed microbiological changes should be interpreted as associations following the combined protocol rather than as evidence of causation or as effects attributable to any individual component. Participants were included based on availability of paired pre- and post-treatment salivary diagnostic data collected during routine clinical care, and follow-up testing occurred at variable intervals ranging from 25 to 274 days post-treatment as part of routine scheduling. Compliance with the daily oral care regimen and probiotic use was self-reported, and systemic antibiotic use is an important confounding factor that may have contributed substantially to the observed bacterial reductions. This study also focused on oral microbiome outcomes and did not include clinical periodontal endpoints such as probing depth, bleeding on probing, attachment loss, or radiographic measures, which should be included to evaluate the complete clinical picture of patient outcomes. Viral outcomes were assessed qualitatively rather than quantitatively, and fungal assessment was limited primarily to *Candida albicans*. While this study was a “proof of concept” showing that a dedicated microbiome focused protocol can help reduce oral pathogens known to cause severe periodontitis, caries, and other oral health issues, future studies should utilize a controlled study design with standardized follow-up intervals and evaluation clinical periodontal parameters associated with oral health.

In conclusion, the Test, Treat, Repopulate method offers a practical, microbiome-focused framework that integrates salivary diagnostics with phased periodontal intervention and microbial repopulation. In this pre-post clinical cohort, the combined protocol was associated with significant reductions in several periodontal and caries-associated bacterial pathogens. However, because the study lacked a control group, included multiple intervention components, and used variable follow-up intervals, these findings should be interpreted cautiously and require confirmation in larger controlled studies. Future work should determine the relative contribution of each protocol component and evaluate whether microbiological improvements correspond with sustained clinical periodontal benefits.

## Data Availability

The original contributions presented in the study are included in the article/Supplementary Material, further inquiries can be directed to the corresponding author.

## Supplementary material

Supplementary Table S1 Cohort participant data including age, sex, days between the pre- and post-treatment salivary diagnostic tests, and quantitative and qualitative target data. For bacterial and fungal targets, ND indicates the target was not detected or below the limit of quantification for the sample; quantitative data are shown in copies/mL of saliva. For viral targets “+” indicates the target was detected while “-” indicates the target was absent from the sample.
